# Supplementary material for: HIVprotI: an integrated web based platform for prediction and design of HIV proteins inhibitors
Source: J Cheminform. 2018 Mar 9;10:12. doi: 10.1186/s13321-018-0266-y (PMC5845081; doi:10.1186/s13321-018-0266-y)
Supplement: Supplementary file 2 — Additional file 2. Source code of HIVProtI web server. [file 13321_2018_266_MOESM2_ESM.zip › HIVprotI_Source-code/hivprotI/weka-3-6-6/doc/weka/core/Trie.TrieNode.html]

Trie.TrieNode


---


|  |  |  |
| --- | --- | --- |
| **Overview** \| **Package** \| **Class** \| **Tree** \| **Deprecated** \| **Index** \| **Help** \| **Weka's home** | |  |
| **PREV CLASS**   **NEXT CLASS** | **FRAMES**    **NO FRAMES**     **All Classes** |  |
| SUMMARY: NESTED \| FIELD \| CONSTR \| METHOD | DETAIL: FIELD \| CONSTR \| METHOD |  |


---


## weka.core Class Trie.TrieNode

```
java.lang.Object
  javax.swing.tree.DefaultMutableTreeNode
      weka.core.Trie.TrieNode
```

**All Implemented Interfaces:**: java.io.Serializable, java.lang.Cloneable, javax.swing.tree.MutableTreeNode, javax.swing.tree.TreeNode, RevisionHandler

**Enclosing class:**: Trie

---

``` public static class Trie.TrieNode extends javax.swing.tree.DefaultMutableTreeNode implements RevisionHandler ```

Represents a node in the trie.

**Version:**
:   $Revision: 1.2 $

**Author:**
:   fracpete (fracpete at waikato dot ac dot nz)

**See Also:**: Serialized Form

---

| **Field Summary** | |
| --- | --- |
| `static java.lang.Character` | `STOP`             the stop character |

**Fields inherited from class javax.swing.tree.DefaultMutableTreeNode**

`EMPTY_ENUMERATION`


**Constructor Summary**

`Trie.TrieNode(char c)`
  
          initializes the node

`Trie.TrieNode(java.lang.Character c)`
  
          initializes the node


| **Method Summary** | |
| --- | --- |
| `boolean` | `add(java.lang.String suffix)`             adds the given string to its children (creates children if necessary) |
| `java.lang.Object` | `clone()`             creates a deep copy of itself |
| `boolean` | `contains(java.lang.String suffix)`             checks whether a suffix can be found in its children |
| `boolean` | `equals(java.lang.Object obj)`             Indicates whether some other object is "equal to" this one. |
| `Trie.TrieNode` | `find(java.lang.String suffix)`             returns the node with the given suffix |
| `java.lang.Character` | `getChar()`             returns the stored character |
| `java.lang.String` | `getCommonPrefix()`             returns the common prefix for all the nodes starting with this node. |
| `java.lang.String` | `getCommonPrefix(java.lang.String startPrefix)`             returns the common prefix for all the nodes starting with the node for the specified prefix. |
| `java.lang.String` | `getRevision()`             Returns the revision string. |
| `java.lang.String` | `getString()`             returns the full string up to the root |
| `boolean` | `remove(java.lang.String suffix)`             Removes a suffix from the trie. |
| `void` | `setChar(java.lang.Character value)`             sets the character this node represents |
| `int` | `size()`             returns the number of stored strings, i.e., leaves |
| `java.lang.String` | `toString()`             returns the node in a string representation |

**Methods inherited from class javax.swing.tree.DefaultMutableTreeNode**

`add, breadthFirstEnumeration, children, depthFirstEnumeration, getAllowsChildren, getChildAfter, getChildAt, getChildBefore, getChildCount, getDepth, getFirstChild, getFirstLeaf, getIndex, getLastChild, getLastLeaf, getLeafCount, getLevel, getNextLeaf, getNextNode, getNextSibling, getParent, getPath, getPreviousLeaf, getPreviousNode, getPreviousSibling, getRoot, getSharedAncestor, getSiblingCount, getUserObject, getUserObjectPath, insert, isLeaf, isNodeAncestor, isNodeChild, isNodeDescendant, isNodeRelated, isNodeSibling, isRoot, pathFromAncestorEnumeration, postorderEnumeration, preorderEnumeration, remove, remove, removeAllChildren, removeFromParent, setAllowsChildren, setParent, setUserObject`

**Methods inherited from class java.lang.Object**

`getClass, hashCode, notify, notifyAll, wait, wait, wait`

**Field Detail**

### STOP

```
public static final java.lang.Character STOP
```

:   the stop character


**Constructor Detail**

### Trie.TrieNode

```
public Trie.TrieNode(char c)
```

:   initializes the node

    **Parameters:**: `c` - the value of this node

---


### Trie.TrieNode

```
public Trie.TrieNode(java.lang.Character c)
```

:   initializes the node

    **Parameters:**: `c` - the value of this node


**Method Detail**

### getChar

```
public java.lang.Character getChar()
```

:   returns the stored character

    :   **Returns:**: the stored character

---


### setChar

```
public void setChar(java.lang.Character value)
```

:   sets the character this node represents

    :   **Parameters:**: `value` - the character to store

---


### add

```
public boolean add(java.lang.String suffix)
```

:   adds the given string to its children (creates children if necessary)

    :   **Parameters:**: `suffix` - the suffix to add to its children **Returns:**: true if the add operation changed the structure

---


### remove

```
public boolean remove(java.lang.String suffix)
```

:   Removes a suffix from the trie.

    :   **Parameters:**: `suffix` - the suffix to remove **Returns:**: true if this trie changed as a result of the call

---


### contains

```
public boolean contains(java.lang.String suffix)
```

:   checks whether a suffix can be found in its children

    :   **Parameters:**: `suffix` - the suffix to look for **Returns:**: true if suffix was found

---


### clone

```
public java.lang.Object clone()
```

:   creates a deep copy of itself

    :   **Overrides:**: `clone` in class `javax.swing.tree.DefaultMutableTreeNode`
    :   **Returns:**: a deep copy of itself

---


### equals

```
public boolean equals(java.lang.Object obj)
```

:   Indicates whether some other object is "equal to" this one.

    :   **Overrides:**: `equals` in class `java.lang.Object`
    :   **Parameters:**: `obj` - the object to check for equality **Returns:**: true if equal

---


### find

```
public Trie.TrieNode find(java.lang.String suffix)
```

:   returns the node with the given suffix

    :   **Parameters:**: `suffix` - the suffix to look for **Returns:**: null if unsuccessful otherwise the corresponding node

---


### getCommonPrefix

```
public java.lang.String getCommonPrefix()
```

:   returns the common prefix for all the nodes starting with this node.
    The result includes this node, unless it's the root node or a STOP node.

    :   **Returns:**: the result of the search

---


### getCommonPrefix

```
public java.lang.String getCommonPrefix(java.lang.String startPrefix)
```

:   returns the common prefix for all the nodes starting with the node
    for the specified prefix. Can be null if initial prefix is not found.
    The result includes this node, unless it's the root node or a STOP node.
    Using the empty string means starting with this node.

    :   **Parameters:**: `startPrefix` - the prefix of the node to start the search from **Returns:**: the result of the search, null if startPrefix cannot be found

---


### size

```
public int size()
```

:   returns the number of stored strings, i.e., leaves

    :   **Returns:**: the number of stored strings

---


### getString

```
public java.lang.String getString()
```

:   returns the full string up to the root

    :   **Returns:**: the full string to the root

---


### toString

```
public java.lang.String toString()
```

:   returns the node in a string representation

    :   **Overrides:**: `toString` in class `javax.swing.tree.DefaultMutableTreeNode`
    :   **Returns:**: the node as string

---


### getRevision

```
public java.lang.String getRevision()
```

:   Returns the revision string.

    :   **Specified by:**: `getRevision` in interface `RevisionHandler`
    :   **Returns:**: the revision


---


|  |  |  |
| --- | --- | --- |
| **Overview** \| **Package** \| **Class** \| **Tree** \| **Deprecated** \| **Index** \| **Help** \| **Weka's home** | |  |
| **PREV CLASS**   **NEXT CLASS** | **FRAMES**    **NO FRAMES**     **All Classes** |  |
| SUMMARY: NESTED \| FIELD \| CONSTR \| METHOD | DETAIL: FIELD \| CONSTR \| METHOD |  |


---
